# Onion Vinegar Quality Evaluation and its Alleviate Oxidative Stress Mechanism in *Caenorhabditis elegans* Via SKN-1

**DOI:** 10.1007/s11130-022-00959-2

**Published:** 2022-04-19

**Authors:** Jianfeng Wang, Xinhua Liu, Shuqin Hang, Chunxin Cao, Yucheng He, Xiaoming Sun, Rongquan Zheng, Jianfeng Yuan

**Affiliations:** 1 Xingzhi College, Zhejiang Normal University, Lanxi, 321100 China; 2 Jinhua Academy of Agricultural Sciences, Jinhua, 321027 China; 3 Key Laboratory of Wildlife Biotechnology and Conservation and Utilization of Zhejiang Province, Zhejiang Normal University, Jinhua, 321004 China

**Keywords:** Onion vinegar, Oxidative stress, Antioxidant enzyme, *Caenorhabditis elegans*, SKN-1

## Abstract

Recently, there has been renewed interest in biorefining of agricultural onion into functional products. In this study, onion vinegar (OV) are prepared by a two-stage semi-continuous fermentation method, and its content of total flavonoids (3.01 mg/mL) and polyphenols (976.76 *μ*g/mL) is superior to other commercial vinegars. OV possesses a high radical scavenging activity and enhances the antioxidant enzyme activities *in vivo*, alleviating intracellular oxidative stress in *Caenorhabditis elegans*. Treated by OV, the 1,1-diphenyl-2-picryl-hydrazyl radical (DPPH·), diammonium 2,2′-azino-bis (3-ethylbenzo thiazoline-6-sulfonic acid) (ABTS^+^·) and 2-phenyl-4,4,5,5- tetramethylimidazoline-1-oxyl 3-Oxide (PTIO·) free radicals clearance rates are 88.76, 98.76 and 90.54%, respectively *in vitro*. Whereas the glutathione peroxidase (GSH-Px), superoxide dismutase (SOD) and catalase (CAT) enzyme activities in *C. elegans* reach 271.57, 129.26, and 314.68%, respectively. Using RNAi and RT-PCR, it has been further confirmed that OV modulates transcription factor SKN-1, the nuclear factor erythroid 2-related factor 2 (Nrf2) homologous, in *C. elegans*, enhancing the resistance of *C. elegans* against sodium arsenite stress. Lifespan analysis reveals that 1 mL OV extends the maximum lifespan of the nematode to 26 days. Evidence is presented which shows that OV increases the lifespan of *C. elegans* by activating the SKN-1 signaling pathway. Overall, the OV is a well functional condiment, enhancing the value-added of onion.

**Supplementary Information:**

The online version contains supplementary material available at 10.1007/s11130-022-00959-2.

## Introduction

Agricultural waste, accounting for over 30% of worldwide agricultural products, is an ideal resource for fermentable carbohydrates. Onions are a major agricultural product owing to their beneficial medicinal [1] and nutritional effects [2]. In 2017, global production of onion increased to 97.8 million tons, while China’s production was 24.34 million tons, accounting for over 24% of the world’s output [3]. Although onions and their by-products are rich in carbohydrates and biologically active compounds with high nutritional value, in Europe, 500,000 tons of onion waste are discarded every year, becoming an environmental issue due to its strong aroma and disposal to landfill [4, 5]. Based on the current situation and the value of the onion itself, therefore, researches on bio-refining processes that convert onion or onion waste into functional products are becoming more important, especially in Asia, which accounts for 63.7% of global onion production.

Existing research recognizes the critical role of vinegar with some functional properties such as digestion and appetite stimulation, antioxidant, fatigue recovery, lipid-lowering effects, and blood pressure regulation [6, 7]. Onion vinegar (OV) has a very interesting potential as a new functional condiment due to its particular composition, including minerals, amino acids and organic acid, and specific physiological properties [8, 9]. They are now well established from a variety of studies, hence the establishment of agricultural OV has positive significance and valorize onion by-products. Previous research, although, shows OV has antioxidant effect *in vitro* [2, 10, 11], the molecular mechanism of OV to alleviate oxidative stress *in vivo* should been comprehensively investigated.

*C. elegans* is a well-established model in genetics, which possesses a homology to 40% of the human-being genome, especially 12 signaling pathways are confirmed to be same as those of the human [12]. Currently, *C. elegans* is used to study various biological processes, including not only cell polarity, cell signaling, cell cycle, gene regulation, senescence, autophagy, and metabolic processes, but also biological effects of natural compounds [13, 14]. SKN-1, the Nrf2 ortholog in *C. elegans*, is a main transcription factor that has a pivotal role in the oxidative stress response, cellular homeostasis, and organismal lifespan [15]. When SKN-1 is activated by oxidative stress, then it translocates into the nucleus where it binds to the antioxidant responsive element in the promoter regions of *e.g*., antioxidative or protective genes [16]. It has been discussed, that Xiangxi flavor vinegar inhibit apoptosis in *C. elegans* probably by scavenging ROS and increasing the activities of antioxidant enzymes like GSH-Px, SOD, and CAT [17], which encourages us to investigate whether OV might alleviate oxidative stress and extend lifespan in *C. elegans* by inducing transcription factor SKN-1.

Herein, OV, prepared by a two-stage semi-continuous fermentation, is evaluated quality *e.g*., total flavonoids, polyphenols, and organic acid, against commercial vinegar. For clarification the alleviation of oxidative stress mechanism, we explore the effects of OV on antioxidantion *in vitro* and *in vivo*. Furthermore, lifespan of nematodes and the potential mechanisms underlying OV induced signal pathway are investigated.

## Materials and Methods

The materials and methods are presented as supplementary material.

## Results and Discussion

### Manufacture and Characteristics of OV

Vinegar, as a traditional acidic condiment, is mainly manufactured through solid fermentation, however, the process is relatively time causing. Here, we produced a semi-continuous fermentation for OV manufacture (Supplementary Fig. 1). The “charge-discharge” procedure was taken out for three cycles. Eventually, the *Y*_A/E_ was 76.71%, and the productivity and *q*_p_ of OV were 17.73 g/(L·d) and 20.51 h^−1^, which was more high than fed-batch process [9]. Considering the rich carbohydrates and various nutrients, onion is a promising source to manufacture the functional condiment. However, there are potential difficulties associated with the certain problems, *i.e*., the low sugar content of onion [18]. In this research, it has been well solved by using onion JHNY3352 (26.53% reducing sugar).

The total flavonoid and polyphenol contents of OV and commercial vinegars were shown in Supplementary Fig. 2, where the flavonoids and polyphenols of OV were 3.01 mg/mL and 976.76 μg/mL, respectively. Notably, during OV manufacture, the total flavonoid contents were reduced by 12.24%, indicating a certain degradation effect on onion flavonoid by *Acetobacter pasteurianus* CICC 20001. However, the situation of total polyphenol contents was different. It was lower than HENGSHUN^®^ (1768.87 *μ*g/mL) and SHUITA^®^ (1487.56 *μ*g/mL). The total flavonoid content of OV was significantly higher than that of commercial vinegar, especially higher than that of vinegar beverage (LAOHENGHE^®^, WASONT^®^, and RUITAI^®^), which was related to the onion itself contained a large amount of flavonoids. But the total polyphenol content was only equal to that of vinegar beverage, which was only 50% of that of traditional vinegar (HENGSHUN^®^ and SHUITA^®^). Overall, OV has higher nutrient content (flavonoid and polyphenol) than common commercial vinegars.

The content of organic acids in OV was determined by HPLC, and the results were shown in Supplementary Table 1. Acetic acid, 40.63 ± 1.97 g/L, accounted for 86.21% of titratable acid, while citrate (5.92 ± 0.12 g/L) and malic acid (0.58 ± 0.03 g/L) accounted for 12.56 and 1.23%. The lactate and succinate were not detected. According to the literature, the amount of organic acids in vinegar was independent of sensory performance, but the ratio of acetic acid to total organic acid (A/T) was highly correlated with taste [19]. The A/T ratio of OV was over 0.86 and, therefore, had a good taste. The quality evaluation displays the advantages of OV to the commercial vinegars. Several studies reveal that the functional ingredients in onion, especially flavonoids, polyphenols and organosulfur, have the healthcare effects [20], indicating the beneficial roles of OV.

### Antioxidant Properties of OV

Studies have shown that vinegar has good antioxidant properties [17, 21]. Combined with the characteristics of OV, we now subject it to extensive investigation on antioxidant properties *in vitro* and *in vivo*.

Considering the stability and sensitivity, DPPH·, ABTS^+^·, and PTIO· are selected to detect the free radical scavenging ability *in vitro*. As can be seen in Supplementary Fig. 3a, DPPH· relative clearance of OV was 88.76%. Among the commercial available vinegars, however, the DPPH· clearance activity by HENGSHUN^®^ was the highest, at 62.64%. In Supplementary Fig. 3b, the ABTS^+^· clearance activity of OV was 98.76%. However, RUITAI^®^ had more clearance activity on ABTS^+^· than other commercial vinegars, up to 89.64%. PTIO· radical scavenge activity was, up to 90.54%, much higher than others (Supplementary Fig. 3c). In conclusion, OV has exhibited a strong advantage in free radicals scavenging than the commercial vinegars *in vitro*.

The antioxidant enzyme activities *in vivo*, therefore, have been assayed using *C. elegans* N2 or RNAi conditions *in vivo* (Supplementary Fig. 3d). Compared to control, the antioxidant enzymes activities of *C. elegans* N2 were increased, except the RNAi sample group (*P* < 0.05). Particularly, the GSH-Px and CAT activities were increased up to 2.0 ~ 2.7 times than control by commercial available vinegars (*P* < 0.01), reaching 271.57 and 314.68%, respectively. However, there was no significant difference in the SOD enzyme activity between the test group and control. In contrast, all the antioxidant enzyme activities of the RNAi group, treated with the OV, were decreased to 76.78 (GSH-Px), 91.56 (SOD) and 79.35% (CAT). The obtained results illustrated that OV enhanced the GSH-Px and CAT enzyme activities of *C. elegans*, while other vinegars depended on increase the GSH-Px enzyme activity.

SOD and CAT, two major antioxidant enzymes in *C. elegans*, have the function to scavenge superoxide free radicals and H_2_O_2_, inducing oxidative damage to biomolecules and resulting in the damage of tissue function [22]. But when *skn-1* gene was silenced, GSH-Px, SOD and CAT activities were decreased, indicating that in *C. elegans*, the antioxidant enzyme activity was regulated by *skn-1* gene *in vivo*.

### OV Modulate Stress Resistance of *C. elegans*

**Fig. 1:**
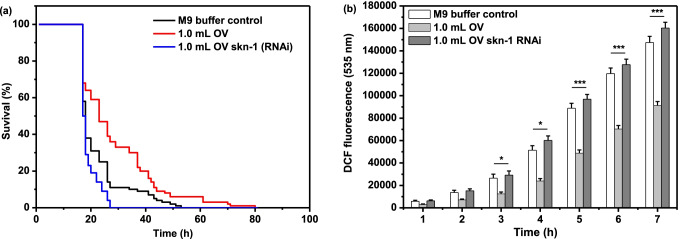
Stress resistance of *C.elegans* by OV (**a**) Suvival of worms assay: L4 synchronized nematodes were treated by 1 mL OV or M9 buffer for 16 h and then were cultured with 5 mM sodium arsenite. (**b**) DCF fluorescence assay *in vivo.* Data are the mean ± SD; * *P* < 0.05; *** *P* < 0.001

The antioxidative active factors upon exogenous stimulation of *C. elegans* was reported to reduce oxidative damage *in vivo*, wherein they found that treating with chlorophyll could effectively prolong the survival of *C. elegans* under naphthoquinone juglone oxidative stress and increase the *sod-3* expression [23]. This motivate us to speculate whether OV also can alleviate oxidative stress in *C. elegans*. We induced oxidative stress in *C. elegans* (N2 and RNAi) with 5 mM sodium arsenite, and then treated with 1.0 mL OV. The survival of the nematodes was monitored. As shown in Fig. 1a. Pretreatment with 1 mL OV significantly prolonged the survival of the *C. elegans* N2 by 52.83%, indicating an increased stress tolerance for sodium arsenite by OV. However, due to the silence of *skn-1* gene (RNAi), the nematode lifespan showed a rapid downward trend, which was only 45.28% of the control.

To clarify whether the antioxidative capacity of OV cause a positive effect on the survival rate of *C. elegans* under thermal stress, we used the fluorescence probe, H_2_DCF-DA, to detect reactive oxygen species (ROS). Once H_2_DCF-DA enters the cell, and converts to H_2_DCF, which cannot penetrate the worm’s cell membrane. In addition, H_2_DCF does not fluorescence until that is oxidized to DCF by ROS, and the DCF fluorescence intensity correlates with ROS [17]. In this assay, compared to control group, the DCF fluorescence density of *C. elegans* pretreated by 1 mL OV decreased significantly, while this results were not detected in the control group and RNAi group. In particular, due to the silence of *skn-1* gene and the accumulation of ROS in nematodes, the DCF fluorescence density was rapidly strengthened, reaching 160,321 ± 5,142 at 7 h (Fig. 1b). In this experimental groups, OV showed the positive ability to alleviate oxidative stress both in survival rate and ROS fluorescence intensity. Meanwhile the lifespan of *C. elegans* was prolonged under oxidative stress.

### OV Activate SKN-1 and Prolong *C. elegans* Lifespan

**Fig. 2:**
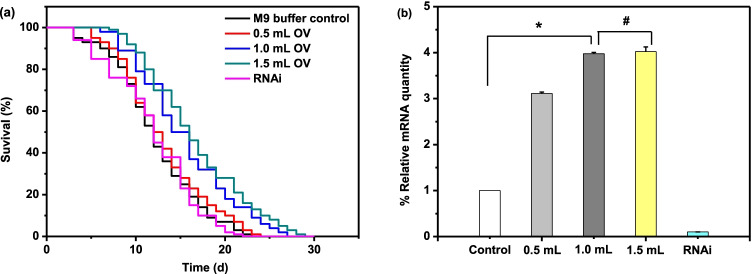
OV prolongs the lifespan of *C.elegans* by activating *skn-1* gene Age-synchronized nematodes were treated with different concentration of OV(0.5 mL, 1 mL, and 1.5 mL) or M9 buffer for 3 d. (**a**) assay the lifespan of *C. elegans* according to the suvival. (**b**) quantitative of *skn-1* gene expression by RT-PCR: effect of OV on gene expression in *C. elegans* normal or *skn-1* RNAi. Data are the mean ± SD; * *P* < 0.05; # No significant difference

Excessive accumulation of ROS is harmful to body, such as promoting apoptosis and aging [17]. However, *C. elegans*, after exogenous stimulation, can activate insulin signal pathway and reduce the intracellular ROS level [23, 24]. To illustrate the antioxidant mechanism, here, we demonstrated a does-dependent effect of OV on lifespan by activating the SKN-1 factor. *C. elegans* treated with 0.5 mL OV prolonged the mean, median and maximum lifespan, but not significant, to 13.04 ± 1.13, 12.00 ± 1.05 and 23, respectively. While 1 and 1.5 mL OV caused an apparent increase of the lifespan against M9 buffer control (*p* value of 0.0078 and 0.0059), however, there was no significant difference between the two groups. It should be noted that there was also no significant difference between RNAi and control group (Fig. 2a, Supplementary Table 2).

In this experiment, the lifespan prolongation was associated with activation of SKN-1 under normal or RNAi condition, and we confirmed this conclusion by determining the *skn-1* gene expression level. When the *C. elegans* was treated by 1 mL OV, the mean, median and maximum lifespan increased by 26.99, 23.02 and 18.18%, respectively, meanwhile, the *skn-1* gene expression level was increased by 2.97 times than control (Fig. 2b). During this process, the longevity effect of OV was not observed in RNAi with the silence of the SKN-1 factor. In the RNAi group, it had a median lifespan of 11.45 ± 1.27 days, while the maximum lifespan was only 21 days.

**Fig. 3:**
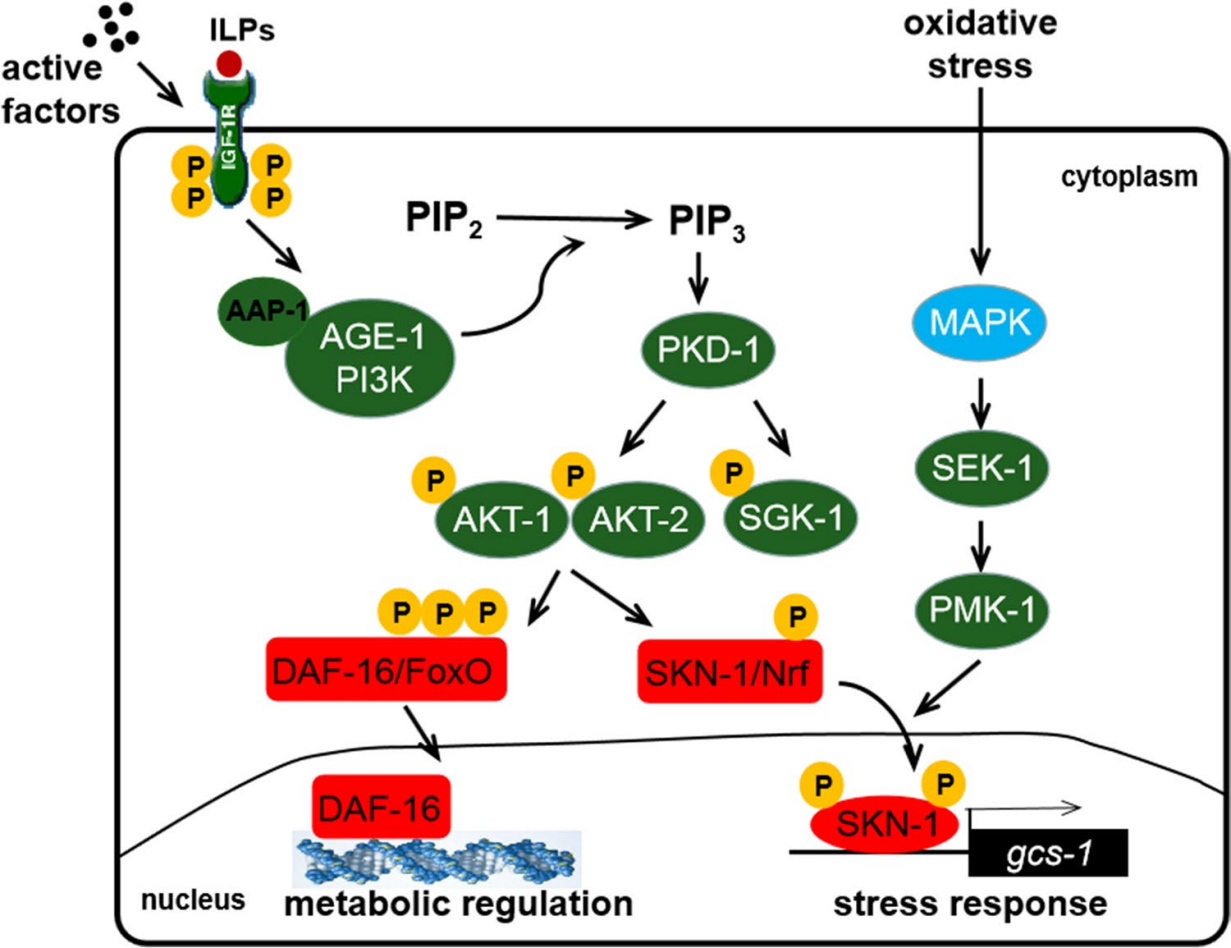
Signal pathways related to antioxidative stress of nutritional active factors in *Caenorhabditis elegans.* The insulin signal pathway components are colored green, and molecules that either antagonize IIS or are antagonized by IIS are colored red. Abbreviations: ILPs, insulin-like peptides; PI3K, phosphoinositide 3-kinase; PIP_2_, phosphatidylinositol 4,5-bisphosphate; PIP_3_, phosphatidylinositol 3,4,5-trisphosphate

It has been found that the *daf-16* gene, homology of FoxO in human, is quite active in most cell *in vivo*, which is associated with *C. elegans* longevity [24, 25]. However, it has also been shown that the extended lifespan of *C. elegans* depends on SKN-1, but not DAF-16 [13, 15]. From the above findings, we can see that the active factors with antioxidative stress capabilities are generally achieved through insulin signaling regulating *daf-16* or activation of *skn-1*. As shown in Fig. 3, insulin interacts with the DAF-2/insulin receptor to activate AGE-1 / PI3K and regulate the activities of multiple downstream genes, including *daf-16* / *FoxO* and *skn-1* transcription factors, enhancing oxidative stress resistance ability of *C. elegans* [26].

SKN-1 is distantly related to the mammalian Nrf proteins and induces detoxification gene transcription, such as *gcs-1*, *sod-1*, *sod-3* [27]. In this study, RNAi and RT-PCR have been used to investigate the role of *C. elegans skn-1* gene in resistance to oxidative stress. We observed that *C. elegans* (RNAi) reduced its resistance ability to oxidative stress due to the silencing of *skn-1* gene, and the fluorescence intensity increased with time, reaching the peak at 7 h. However, the situation was completely opposite in the OV treated group. One ml of OV can improve the activity of antioxidant enzymes and enhance the resistance to oxidative stress, so as to prolong the lifespan of *C. elegans*. This was mainly because OV activated the expression of *skn-1* gene.

## Conclusions

In conclusion, we set up a semi-continuous OV manufacture process and extensively investigate the quality and its alleviate oxidative stress mechanism. We have confirmed that OV alleviate oxidative stress by activating *skn-1* gene, the Nrf homolog, in *C. elegans*. This shows that OV has strong potential for antioxidant and anti-aging, which is a functional condiment. In addition, OV manufacture can not only enrich people’s condiment taste, but also enhance the value-added of onion.

## Supplementary Information

ESM 1 (DOC 156 kb)

## Data Availability

Data is available upon request.
