# Supplementary material for: Onion Vinegar Quality Evaluation and its Alleviate Oxidative Stress Mechanism in Caenorhabditis elegans Via SKN-1
Source: Plant Foods Hum Nutr. 2022 Apr 19;77(2):206–11. doi: 10.1007/s11130-022-00959-2 (PMC9192463; doi:10.1007/s11130-022-00959-2)
Supplement: Supplementary file 1 — (DOC 156 kb) [file 11130_2022_959_MOESM1_ESM.doc]

**Materials and methods**

**Materials**

Onion (*Allium cepa* L.) JHNY3352 provided by Jinhua Academy of Agricultural Sciences. Commercial vinegars were purchased including HENGSHUN® (aromatic vinegar), SHUITA® (aged vinegar), LAOHENGHE® (rose rice vinegar), WASONT® (apple cider vinegar), and RUITAI® (persimmon vinegar). *Saccharamyces cerevisiae* ATCC 9763 was purchased from ATCC maintained on YM medium plate, and *Acetobacter pasteurianus* CICC 20001 was purchased from Shanghai Bioresource Collection Center, China. *Caenorhabditis elegans* N2 was purchased from Caenorhabditis Genetics Center (CGC), University of Minnesota. Glutathione Peroxidase (GSH-Px) assay kit (A005-1-2), Superoxide Dismutase (SOD) assay kit (WST-1 method, A001-3-2), and Catalase (CAT) assay kit (Visible light, A007-1-1) were all purchased from Nanjing Jiancheng Bioengineering Institute, China. Other reagents were all purchased from Sangon Biotech (Shanghai) Co., Ltd, China.**OV manufacture**

Preparation of onion juice from onions JHNY3352 with the 34.74% carbohydrate content (wet weight) composed mainly of glucose (20.70%), fructose (4.34%), sucrose (8.39%), galactose (1.27%), mannose (0.03%), xylose (0.005%), and arabinose (0.004%). The sugar content of onion juice was adjusted to 14 °Brix, pH 6.2, and fermented for 48 h at 30 °C by *S. cerevisiae* ATCC 9763, with the condition of inoculum 5% (v/v), 100 rpm, and 0.1, vvm, followed by *A. pasteurianus* CICC 20001 for OV manufacture with the initial onion ethanol 5% (v/v), inoculum 10% (v/v) at 28 °C, 300 rpm, 0.2 vvm, for 72 h. The semi-continuous fermentation proceeded to “charge-discharge” [1], which was carried out by removing 75% of fermentation broth and adding the same amount of ethanol-fermented onion juice for 3 cycles. During proceeding, biomass was measured by a spectrophotometer at 600 nm, corresponding to cell dry weight (DCW). According to the instructions, ethanol was determined by Biovision kit (Catalog #: K620). Acetic acid was determined by titrate acidity.

**Total flavonoids, polyphenols, and organic acids determination**

Total flavonoid of the samples was measured by spectrophotometry at 510 nm [2]. Briefly, in a 10 mL plug test tube, OV samples, 0.3 mL 10% NaNO_2_ were mixed and reaction for 6 min. Subsequently, 0.3 mL of 10% Al(NO_3_)_3_ was added to the reaction mixture for another 6 min. At last, 4 mL NaOH solution (4%) was added, and the capacity was fixed with 30% ethanol, mixed well. Absorbance values of 510 nm were measured, and lutin serves as the standard control. The total polyphenols content was determined with the Folin-Ciocalteu method as previously described by ISO 14502-1 [3]. 1 mL of diluted OV was mixed with 5 mL of 10% (v/v) Folin-Ciocalteu phenol reagent and 4 mL of 7.5% (w/v) Na_2_CO_3_, respectively. The mixture was left at ambient temperature for 1 h before the absorbance was measured at 765 nm, using gallic acid monohydrate (ACS reagent, ≥98.0%) as a standard. The organic acids were determined by HPLC. Conditions: chromatographic column, Agilent ZORBAX Eclipse Plus C18 (2.1×50 mm, 1.8 μm), column temperature of 30 °C, flow rate of 0.5 mL·min^-1^, injection volume of 10 μL, and mobile phase: gradient elution of methanol (A)-0.1% aqueous formic acid (B) wavelength of 210 nm.

**Free radical scavenging *in vitro***

0.3 mL sample and 2.7 mL DPPH solution (dissolved in methanol, 0.5 mM) were mixed, letting it stand and avoid light reaction for 20 min. Taking blank as negative control, the absorbance values at 519 nm were measured, and 2 mM Vc solution was used as positive control [4]. The clearance rate is calculated as follows: Where *A*_0_, *A*_s_ are the absorbance value of blank and samples.

$$\text{DPPH}\cdot\text{ clearance rate }(\%)=\frac{A_{0}-A_{s}}{A_{0}}\times100$$

7.4 mM of ABTS diammonium and 2.6 mM of potassium perbisulfite (K_2_S_2_O_8_) were mixed, standing for 16 h at room temperature in darkness. The mixture was again diluted with phosphate buffer (pH 7.4) to the absorbance value at 734 nm of 0.700±0.002, preparing the ABTS^+^· working solution [5], and then 0.1 mL of sample and 3.9 mL of ABTS^+^· solution were mixed and the absorbance was measured at 734 nm after reaction for 6 min, where *A*_0_, *A*_s_ are the absorbance value of blank and samples.

$$\text{ABTS}^{+}\cdot clearance rate (\%)=\frac{A_{0}-A_{s}}{A_{0}}\times100$$

0.64 mM PTIO· test solution was prepared and diluted with methanol to have an absorbance value of 0.400-0.600 at 557 nm. 0.4 mL of sample and 1.6 mL of PTIO· solution were mixed, and incubated at 37 °C for 30 min. Considering blank as negative control, the absorbance values at 557 nm were measured [6], against 2 mM Vc solution as positive control, where *A*_0_, *A*_s_ are the absorbance value of blank and samples.

$$\text{PTIO}\cdot\text{ clearance rate }(\%)=\frac{A_{0}-A_{s}}{A_{0}}\times100$$

**Maintenance of *C. elegans***

The *C. elegans* N2 was routinely maintained at 20 °C on nematode growth medium (NGM) seeded with *E. coli* OP50 as nutrient as described previously [7]. Age synchronized nematodes were achieved by treating gravid hermaphrodites with bleach (10% sodium hypochlorite: 1 M sodium hydroxide =1:1). The eggs were collected and incubated in buffer M9 for one day up to the embryos hatched at the L1 stage, and then were transferred to new fresh NGM plates until L4 synchronized nematodes were obtained.

**cDNA preparation and quantitative real-time PCR**

Total RNA was extracted using EZ-10 Spin Column Total RNA Isolation Kit (Bio Basic Inc). Double-stranded cDNA was synthesized from total RNA by RNA PCR Kit (AMV) ver. 3.0 (TaKaRa Bio Inc) according to the manufacturer. The products were quantified via real-time PCR with StepOnePlusTM Real-Time PCR System (Applied Biosystems, USA) using primers SKN-1_F: 5’-TCCACCAGCATCTCCATTCG-3’ and SKN-1_R: 5’-CTCCATAGCACATCAATCAAGTCG-3’ to detect the *skn-1* gene expression. The glyceraldehyde triphosphate dehydrogenase (*gpd-2*) was used as a reference with the primers GPD-2_F: 5’-GCATCATC ATTTTCAATTTTCC-3’ and GPD-2_R: 5’-GACGAAACATGTGCAGTAAGC-3’ [8].

**Silence the target *skn-1* gene**

RNAi at target *skn-1* gene was carried out as described. The *skn-1* RNAi plasmid consisted of a full length SKN-1 cDNA (1568 bp insert, Chromosome IV, NC_003282.8, sequence targeted including exons 4, 5 and 6 of *skn-1*a), subcloned into pL4440 [9]. The *E. coli* HT115(DE3), harboring confirmed *skn-1* RNAi plasmid, was cultured in LB liquid (containing 100 μg/mL ampicillin, 50 μg/mL tetracycline, and 100 μmol/L of IPTG) at 37 °C for 4 h. Subsequently, the resulted *E. coli* HT115 was collected, suspended in M9 buffer, and applied on NGM plates (containing 100 μg/mL ampicillin, 50 μg/mL tetracycline, and 100 μmol/L IPTG). The synchronized wild-type nematodes (N2) L1 larvae were placed at 20 °C on *E. coli* HT115 NGM plate that expressed dsRNA of either *skn-1*a or control for 46 h, until they reached the L4 stage.

**Antioxidant enzymes assay *in vivo***

1.0 mL OV or M9 buffer control was spread on NGM plate. The age synchronized L4 nematodes normal or RNAi conditions were transferred and incubated at 20 °C for 48 h. Then 0.1±0.05 g nematodes were collected and transferred to 1.5 mL sterile Eppendorf tubes containing a 9-fold volume of 0.9% saline. Nematodes were sonicated using an ultrasonic cell crusher (Vosin BL92-IIDL, Wuxi, China) and centrifuged at 2500 r/min for 15 min. The supernatant was transferred to a new sterile Eppendorf tube and maintained at 60 °C in water bath for 15 min. The second centrifugation was carried out at 4000 r/min for 20 min. The resultant supernatant was used for GSH-Px, SOD, and CAT activities determination, according to the manufacturer’s instructions of the kits (Nanjing Jiancheng Bioengineering Institute, Nanjing, China).

**Assessment of stress resistance**

The age-synchronized nematodes, N2 nematodes normal or RNAi conditions, were incubated on NGM plates at 20 °C for 2 days supplied with 1.0 mL OV or M9 buffer control. After incubation, the nematodes were collected and divided into two parts. One portion were transferred to M9 buffer containing 5 mM sodium arsenite and 1×10^9^ OP50/mL as a food source. Survival of the nematodes was monitored regularly during day time using touch-provoked movement [10]. Another portion of nematodes were washed in PBST (PBS with 0.1% Tween 20), subsequently, the nematodes were individually transferred into the wells of a 96-well microplate with a clear bottom containing M9 buffer. The final concentration of 50 μM 2,7-dichlorodihydrofluorescein diacetate (H_2_DCF-DA, Sigma) was added, and the plate was sealed against evaporation and thermal stress (37 °C) was applied. The DCF fluorescence was measured by Thermo Scientific Luminoskan (excitation, 485 nm; emission, 535 nm) after the onset of the thermal stress condition [9].

**Lifespan analysis**

Age-synchronized N2 nematodes (normal or RNAi conditions), were incubated with various concentrations of OV or M9 buffer control, and were kept at 25 °C. Survival was examined daily by first touching posterior and then anterior. *C. elegans* that were scored as alive were transferred into fresh incubation medium. Nematodes displaying internal hatching or protruding organs were excluded [11].

**Statistical analysis**

Statistical analysis was carried out using SPSS (ver. 16.0), and the comparisons of differences between the means of the treatments were tested by one-way analysis of variance (ANOVA) at a significance level of P<0.05.

**References**

1. Lee S, Lee JA, Park GG, Jang JK, Park YS (2017) Semi-continuous fermentation of onion vinegar and its functional properties. Molecules 22:1313. <http://doi.org/10.3390/molecules22081313>
2. Tang X, Zhai JM, Ge XF (2021) Comparative study on antioxidant active components of three natural plants for food packaging. Sci Technol Food Ind 42(14):86-92. http://doi.org/[10.13386/j.issn1002-0306.2020110284](http://dx.doi.org/10.13386/j.issn1002-0306.2020110284)
3. Thuengtung S, Ogawa Y (2020) Comparative study of conventional steam cooking and microwave cooking on cooked pigmented rice texture and their phenolic antioxidant. Food Sci Nutr 8(2):965-972.

http://doi.org/10.1002/fsn3.1377

1. Li XC (2018) Comparative study of 1,1-Diphenyl-2-picryl-hydrazyl radical (DPPH·) scavenging capacity of the antioxidant xanthones family. Chemistryselect 46(3):13081-13086. http://doi.org/[10.1002/slct.201803362](https://doi.org/10.1002/slct.201803362)
2. Li XC, Ouyang XJ, Cai RX, Chen DF (2019) 3,8-Dimerization enhances the antioxidant capacity of flavonoids: evidence from acacetin and isoginkgetin. Molecules **24**:2039. <http://doi.org/10.3390/molecules24112039>
3. Li XC (2017) 2-Phenyl-4,4,5,5-tetramethylimidazoline-1-oxyl 3-oxide (PTIO·) radical scavenging: a new and simple antioxidant assay *in vitro*. J Agric Food Chem 65(30):6288-6297. <http://doi.org/10.1021/acs.jafc.7b02247>
4. Gu QL, Zhang Y, Fu XM, Lu ZL, Yu Y, Chen G, Ma R, Kou W, Lan YM (2020) Toxicity and metabolism of 3-bromopyruvate in *Caenorhabditis elegans*. J Zhejiang Univ Sci B 21(1):77-86. <http://doi.org/10.1631/jzus.B1900370>
5. Nargund AM, Fiorese CJ, Pellegrino MW, Deng P, Haynes CM (2015) Mitochondrial and nuclear accumulation of the transcription factor ATFS-1 promotes OXPHOS recovery during the UPR^mt^. Mol Cell 58(1):123-133. http://doi.org/[10.1016/j.molcel.2015.02.008](https://doi.org/10.1016/j.molcel.2015.02.008)
6. Dehghan E, Zhang YQ, Saremi B, *et al*. (2017) Hydralazine induces stress resistance and extends *C. elegans* lifespan by activating the NRF2/SKN-1 signalling pathway. Nat Commun 8(1):2223.

<http://doi.org/10.1038/s41467-017-02394-3>

1. Havermann S, Humpf HU, [Wätjen](https://www.sciencedirect.com/science/article/pii/S0367326X16301423#!) W (2016) Baicalein modulates stress-resistance and life span in *C. elegans* via SKN-1 but not DAF-16. Fitoterapia 113:123-127. [http://doi.org/10.1016/j.fitote.2016.06.018](https://doi.org/10.1016/j.fitote.2016.06.018)
2. Havermann S, Rohrig R, Chovolou Y, Humpf HU, [Wätjen](https://pubmed.ncbi.nlm.nih.gov/?term=Wätjen+W&cauthor_id=23339711) W (2013) Molecular effects of baicalein in hct116 cells and *Caenorhabditis elegans*: activation of the Nrf2 signaling pathway and prolongation of lifespan. J Agric Food Chem 61(9): 2158-2164. [http://doi.org/](https://doi.org/10.1016/j.fitote.2016.06.018)[10.1021/jf304553g](https://doi.org/10.1021/jf304553g)

**Supplementary Fig.1** Semi-continuous OV fermentation with *A.pasteurianus* CICC 20001. black square (■) DCW; red circle (●) ethanol; blue triangle (▲) titratable acidity

**Supplementary Fig.2** **Contents of OV and commercial vinegars** (a) total flavonoid contents; (b) total polyphenol contents; 1 onion ethanol; 2 OV; 3 HENGSHUN®; 4 SHUITA®; 5 LAOHENGHE®; 6 WASONT®; 7 RUITAI®

**Supplementary Fig. 3 Antioxidant activities assay *in vitro* and *in vivo*. Part a: free radical scavenging** (a) DPPH·, (b) ABTS^+^·, (c) PTIO·, 1 vitamin C (2 mM, relative activity 100%); 2 OV; 3 HENGSHUN®; 4 SHUITA®; 5 LAOHENGHE®; 6 WASONT®; 7 RUITAI®. **Part b: the antioxidant enzymes activity assay** (d) GSH-Px, SOD, and CAT activities in *C.elegans*. 1 M9 control; 2 RNAi with OV; 3 acetic acid; 4 OV; 5 HENGSHUN®; 6 SHUITA®; 7 RUITAI®；* P<0.05; ** P<0.01

**Supplememtary Table 1 Analysis of organic acid content in OV**

| Main organic acid | Concentration (g/L) | Percentage (%) |
| --- | --- | --- |
| Acetic acid | 40.63±1.97 | 86.21 |
| Citric acid | 5.92±0.12 | 12.56 |
| Malic acid | 0.58±0.03 | 1.23 |
| Lactic acid | - | - |
| Succinic acid | - | - |

**Supplementary Table 2 Means, medians, and maximum survival of *C. elegans***

|  | Means  (day) | Median  (day) | Maximum  (day) | Number of nematodes | *p* value vs. control |
| --- | --- | --- | --- | --- | --- |
| M9 buffer control | 12.19±1.11 | 11.38±1.12 | 22 | 150 | - |
| 0.5 mL OV | 13.04±1.13 | 12.00±1.05 | 23 | 149 | 0.0580 |
| 1.0 mL OV | 15.48±0.93 | 14.00±0.89 | 26 | 150 | 0.0078 |
| 1.5 mL OV | 16.62±0.84 | 15.21±1.07 | 28 | 148 | 0.0059 |
| RNAi | 11.93±1.08 | 11.45±1.27 | 21 | 149 | - |
